# Associations of job-related emotional exhaustion and career decision regret with quiet quitting among private sports facility employees: a cross-sectional mediation study

**DOI:** 10.3389/fpsyg.2026.1968120

**Published:** 2026-10-21

**Authors:** Faik Orhun Tapşın, Fuat Orkun Tapşın, Gökhan Çakmak

**Affiliations:** 1 School of Physical Education and Sports, Dicle University, Diyarbakır, Türkiye; 2 Institute of Health Sciences, Kocaeli University, Kocaeli, Türkiye; 3 Sports Sciences Faculty, Kocaeli University, Kocaeli, Türkiye

**Keywords:** career decision regret, employee well-being, job-related emotional exhaustion, private sports facilities, quiet quitting

## Abstract

**Aim:**

This study examined the associations of job-related emotional exhaustion and career decision regret with quiet quitting among private sports facility employees and tested whether career decision regret statistically mediates the association between job-related emotional exhaustion and quiet quitting.

**Method:**

In this cross-sectional survey, administrative staff, fitness trainers, personal trainers, Pilates instructors, and yoga instructors aged 19 and over working in private sports facilities in four districts of Sakarya and Kocaeli, Türkiye, were recruited by convenience sampling. Of 700 questionnaires obtained face-to-face on a voluntary basis, 646 valid questionnaires were analyzed. The data collection instruments were a demographic information form, the Job-Related Emotional Exhaustion Scale, the Quiet Quitting Scale, and the Career Decision Regret Scale. Mann–Whitney U, Kruskal–Wallis, Spearman correlation, and hierarchical regression analyses were applied, and statistical mediation was tested using PROCESS Macro (Model 4) with a 95% bootstrap confidence interval.

**Results:**

In secondary comparisons, men reported higher career decision regret than women (*p* < 0.001), whereas job-related emotional exhaustion and quiet quitting did not differ by gender. Married employees reported higher quiet quitting than single employees. Part-time employees reported higher job-related emotional exhaustion, and full-time employees reported higher career decision regret. All three scores differed across age and duration-of-employment groups in non-linear patterns. Job-related emotional exhaustion was positively correlated with quiet quitting (*r* = 0.474, *p* < 0.001) and career decision regret (*r* = 0.253, *p* < 0.001), and quiet quitting was positively correlated with career decision regret (*r* = 0.344, *p* < 0.001). In the hierarchical regression, age, job-related emotional exhaustion, and career decision regret statistically predicted quiet quitting (R^2^ = 0.342). In the PROCESS model, the total (B = 0.5096) and direct (B = 0.4275) associations of job-related emotional exhaustion with quiet quitting were significant, and the indirect association through career decision regret was small but significant (B = 0.0821; 95% CI [0.0481, 0.1218]), corresponding to about 16.1% of the total association as a descriptive ratio (0.0821/0.5096).

**Conclusion:**

Among private sports facility employees, job-related emotional exhaustion was positively associated with quiet quitting, and career decision regret showed partial and modest statistical mediation of this association. These cross-sectional findings are consistent with, but do not establish, the proposed process; they cannot determine temporal or causal ordering and apply only to the sampled private sports facilities.

## Background

The sports sector has become one of the service areas that has grown globally in recent years and whose economic value is steadily increasing (Zhai et al., 2024). Employees working in different structures such as sports clubs, fitness centers, sports federations, recreation businesses, and private sports organizations play a critical role in ensuring the sustainability and service quality of the sector (Hums and McLean, 2013; Moon et al., 2022; Dugalić and Krsteska, 2013). However, work in sport settings can place significant psychological burdens on employees due to intense human interaction, performance pressure, irregular working hours, customer expectations, and constantly changing organizational demands (Weight et al., 2021; Mercer et al., 2025). Evidence from athlete samples also indicates that the quality of coach–athlete relationships is associated with competition anxiety, illustrating how interpersonal relationships may shape psychological responses in demanding sport environments (Şahinler et al., 2025). This situation may be related not only to the job performance of employees but also to their attitudes towards their jobs and professions (Bhatti and Alnehabi, 2023; Yang and Qin, 2022).

The quality of sports services largely depends on employee motivation, commitment, and voluntary participation in organizational processes (Xie, 2005; Chiu et al., 2020). Related sport research indicates that coach behaviors are associated with athlete self-efficacy, highlighting the potential importance of relationship-based leadership in shaping psychological resources within sport environments (Öztürk et al., 2026). However, recent transformations in working life have been accompanied by significant changes in employees’ perspectives on their jobs (Thompson and Parent, 2021). Particularly with the emergence of the concept of quiet quitting, researchers have begun to focus on why employees reduce their extra efforts toward the organization and prefer to perform only their basic duties (Atiq et al., 2025; Boz et al., 2023; Öztürk et al., 2023). Quiet quitting refers to remaining in one’s job while deliberately limiting effort to the minimum requirements of the role and withholding discretionary contributions (Galanis et al., 2023; Atiq et al., 2025; Öztürk et al., 2023). Conceptually, it is related to but distinct from neighboring constructs. Work disengagement denotes a general lack of energy for and involvement in work, and psychological withdrawal covers a broad range of mental distancing from work, whereas quiet quitting denotes a deliberate restriction of effort to formally required tasks. Reduced organizational citizenship behavior concerns only the extra-role part of performance, whereas quiet quitting also involves reduced initiative and motivation within the role, as reflected in the detachment, lack of initiative, and lack of motivation components of the Quiet Quitting Scale (Galanis et al., 2023). Turnover intention refers to the intention to leave the organization, whereas employees who quietly quit remain in their jobs. Considering the human-centered nature of private sports facilities, quiet quitting may create organizational problems.

One of the factors that may be associated with quiet quitting is job-related emotional exhaustion (Gökkaya, 2024). Emotional exhaustion refers to the decrease in an individual’s emotional resources and feeling psychologically exhausted as a result of the constant demands they face in their work life (Bocheliuk et al., 2020; Özdemir and Kerse, 2020). It is regarded as the core component of burnout rather than the burnout syndrome as a whole (Seidler et al., 2014). The instrument used in this study (Wharton, 1993; Günay, 2021) measures job-related emotional exhaustion only, and the term is used accordingly throughout the manuscript. Individuals working in sports facilities are expected to maintain the emotional labor required for service delivery while simultaneously meeting performance expectations (Lee, 2023; Jones, 2002). This situation may be accompanied by a depletion of employees’ emotional resources and a decrease in their energy for work (Weight et al., 2021).

## Theoretical framework

The proposed model is grounded in Conservation of Resources (COR) theory, according to which individuals strive to obtain, retain, and protect valued resources, and resource loss is more salient than resource gain (Hobfoll et al., 2018). From this perspective, sustained emotional demands may deplete employees’ psychological resources, and employees whose resources are depleted may adopt a defensive posture and limit further investment of effort in order to protect what remains (Hobfoll et al., 2018). Consistent with this reasoning, emotional exhaustion has been examined within a COR framework in relation to organizational commitment and turnover (Lapointe et al., 2011) and has been linked to counterproductive work behaviors (Bolton et al., 2012). Restricting effort to the minimum requirements of the role, that is, quiet quitting, can therefore be viewed as one possible resource-conserving response to job-related emotional exhaustion. This is a theoretical proposition; the present cross-sectional data can show only whether the observed associations are consistent with it.

Understanding the association between job-related emotional exhaustion and quiet quitting may also require examining employees’ evaluations of their occupational choice. In a sample of healthcare workers, Gökkaya (2024) tested quality of work life, not career decision regret, as the mediator of this association. In this context, career decision regret refers to an individual’s reassessment of their career choices and their assessment that a different career path might have yielded more favorable results (Budjanovcanin and Woodrow, 2022). In the present study, the regretted decision is the individual’s choice of occupation or career path, as assessed by the Career Decision Regret Scale (Erdurcan and Kırdök, 2017); it is not the decision to work at a particular facility. Career decision regret is therefore a retrospective evaluation of the occupational choice that is broader than momentary dissatisfaction with the current job. It differs from job dissatisfaction, which is an evaluation of the present job and its conditions, and from turnover intention, which is a forward-looking intention to leave the employer: an employee may regret the occupational choice without intending to leave, and may intend to leave without regretting the occupation. Within COR reasoning, career decision regret may represent a cognitive-evaluative link between depleted resources and minimum-role withdrawal. Employees who feel persistently exhausted may, over time, re-evaluate their job satisfaction, career expectations, and professional experiences (Polat and Yeşil, 2025; Bostan et al., 2025), and those who come to regret their occupational choice may see less reason to invest discretionary effort. However, the reverse ordering is also plausible: employees who already regret their career choice may be more vulnerable to emotional exhaustion, and the two may reinforce each other. A cross-sectional design cannot determine the temporal order of these variables. Given the performance expectations and interaction-intensive nature of sports facility work, employees’ evaluations of their occupation may be related to their attitudes and behaviors in the workplace (Wang et al., 2024; Todd and Kent, 2009). Within sport organizations, decision-making represents an important component of professional functioning, with previous research highlighting considerable attention to the individual and occupational characteristics associated with decision-making among sport managers (Eroğlu and Çeviker, 2022).

Although emotional exhaustion, quiet quitting, and career decision regret have been examined in different sample groups, research addressing these variables together among sports facility employees is limited. The private sports facility context adds to the healthcare evidence (Gökkaya, 2024) in several respects. Fitness trainers, personal trainers, and Pilates and yoga instructors work in continuous face-to-face contact with members and perform instructional emotional labour, as they are expected to motivate members throughout each session (Maguire, 2001; Smith Maguire, 2008; Lee, 2023). Work in such facilities may also involve variable shifts, session-based work and pay, part-time contracts, and limited career pathways (Maguire, 2001); in the present sample, 43.2% of participants worked part-time. In addition, the quality of the service depends directly on employee behavior. These features make both resource depletion and re-evaluation of the occupational choice plausible in this setting. The present study does not cover the sports sector as a whole; it is limited to employees of private sports facilities in four districts of the Sakarya and Kocaeli provinces of Türkiye. The aim of this study was to examine the associations of job-related emotional exhaustion and career decision regret with quiet quitting among private sports facility employees and to test whether career decision regret statistically mediates the association between job-related emotional exhaustion and quiet quitting. Differences in the three variables according to gender, marital status, employment type, age, and duration of employment were examined as secondary, exploratory analyses and are not part of the hypothesized model. The findings of this research are expected to contribute to understanding employee behavior in private sports facilities and to inform practices aimed at supporting employee well-being.

## Method

### Research model

This study examined the association between job-related emotional exhaustion and quiet quitting among employees of private sports facilities and tested the statistical mediating role of career decision regret in this association. The research was conducted using a quantitative research approach and has a descriptive, comparative, correlational, and predictive research design based on a cross-sectional survey model. Ethical approval was obtained from the Dicle University Social and Human Sciences Ethics Committee before the study (approval date: 28 April 2026; approval number: 1136557). The study was conducted between May and June 2026. Participants were informed about the purpose of the study and took part voluntarily. The authors obtained written informed consent forms from the participants. All variables were measured by self-report in a single session. Because the design is cross-sectional, it does not allow temporal or causal inference.

### Hypotheses and secondary research question

*H*1: Job-related emotional exhaustion is positively associated with quiet quitting.

*H*2: Job-related emotional exhaustion is positively associated with career decision regret.

*H*3: Career decision regret is positively associated with quiet quitting.

*H*4: Career decision regret statistically mediates the association between job-related emotional exhaustion and quiet quitting.

Secondary research question (exploratory): Do job-related emotional exhaustion, quiet quitting, and career decision regret differ according to gender, marital status, employment type, age, and duration of employment in the sports sector? These comparisons are not part of the hypothesized model.

### Research group

This research was conducted with administrative staff, fitness trainers, personal trainers, Pilates instructors, and yoga instructors working in private sports facilities in the districts of Sapanca, Serdivan, Başiskele, and İzmit in the Sakarya and Kocaeli provinces of Türkiye. These districts were selected because of their high concentration of private sports facilities and the easy access to participants working in different roles. The research was conducted at these private sports facilities at different times during weekdays and weekends.

Participants were approached face-to-face at private sports facilities, informed about the purpose of the study, and data were collected on a voluntary basis. To increase participant diversity, the data collection process was conducted during weekday and weekend mornings, afternoons, and evenings. The study included administrative staff, fitness trainers, personal trainers, Pilates instructors, and yoga instructors who had worked at private sports facilities for at least 1 year. A total of 700 questionnaires were obtained; 54 were excluded because they were incomplete or incorrectly completed, and the remaining 646 questionnaires were included in the analysis (valid-case proportion: 646/700 = 92.3%). This figure is reported as a valid-case proportion and not as a response rate. The research group was reached using a convenience sampling method and consisted of individuals aged 19 and over. Using the G*Power 3.1.9.7 program, a power analysis was conducted, taking into account a moderate effect size (f^2^ = 0.15), a significance level of α = 0.05, and a 95% test power (1 − β = 0.95). As a result, the minimum sample size was calculated to be 107. With 646 participants in the study, the number of participants exceeded the required minimum sample size, and the study’s sample size was deemed sufficient. The question “Have you worked at private sports facilities for at least one year?” was included in the information form, and individuals who answered “yes” were included in the study. Individuals who answered “no” were excluded. This criterion was applied so that the sample would consist of employees with at least 1 year of professional experience in private sports facilities.

Table 1 presents the distribution of the demographic characteristics of the study group. Of the participants, 54.5% (*n* = 352) were female and 45.5% (*n* = 294) were male. Regarding age distribution, 69.2% (*n* = 447) of the participants were aged 19–27 years, 17.3% (*n* = 112) were aged 28–37 years, and 13.5% (*n* = 87) were aged 38 years or older. In terms of marital status, 76.9% (*n* = 497) of the participants were single, while 23.1% (*n* = 149) were married. Regarding the duration of employment in the sports sector, 73.4% (*n* = 474) of the participants had 1–5 years of experience, 11.1% (*n* = 72) had 6–10 years, 9.8% (*n* = 63) had 11–15 years, and 5.7% (*n* = 37) had 16 years or more of experience. In terms of employment type, 56.8% (*n* = 367) of the participants worked full-time, while 43.2% (*n* = 279) worked part-time.

**Table 1:** Demographic characteristics of the research group.

Characteristic	Category	Frequency (f)	Percentage (%)
Gender	Female	352	54.5
	Male	294	45.5
Age	19–27 years	447	69.2
	28–37 years	112	17.3
	38 years and older	87	13.5
Marital status	Married	149	23.1
	Single	497	76.9
Duration of employment in the sports sector	1–5 years	474	73.4
	6–10 years	72	11.1
	11–15 years	63	9.8
	16 years or more	37	5.7
Employment type in the sports sector	Part-time	279	43.2
	Full-time	367	56.8

### Data collection tools

An information form developed by the researchers was used in the study. The information form included questions regarding the participants’ demographic characteristics, such as gender, age, marital status, duration of employment in the sports sector, and type of employment in the sports sector. In addition to the demographic information form, the Job-Related Emotional Exhaustion Scale, Quiet Quitting Scale, and Career Decision Regret Scale were also used in the study.

#### Job-related emotional exhaustion scale

The Job-Related Emotional Exhaustion Scale was developed by Wharton (1993) and adapted into Turkish by Günay (2021). It measures the emotional exhaustion that employees experience in relation to their job, which is the core dimension of burnout, and does not assess the broader burnout syndrome. The scale has a unidimensional structure and consists of six items. It is rated on a 5-point Likert-type scale ranging from “0 = I never feel this way at work” to “4 = I feel this way every day.” Item scores are summed to a total score (possible range: 0–24), with higher scores indicating greater job-related emotional exhaustion. In the Turkish adaptation study conducted by Günay (2021), the Cronbach’s alpha internal consistency coefficient of the scale was determined to be 0.903. In this study, however, the scale’s Cronbach’s alpha value was found to be 0.74.

#### Quiet quitting scale

The Quiet Quitting Scale was developed by Galanis et al. (2023) and adapted into Turkish by Akyazı and Balcı Çelik (2025). The scale consists of nine items and is rated on a 5-point Likert-type scale. Items 4 and 7 are reverse-scored. The original scale has a three-factor structure comprising detachment, lack of initiative, and lack of motivation (Galanis et al., 2023). Higher scores obtained from the scale indicate higher levels of quiet quitting among individuals. In the present study, the scale was evaluated based on the total score (possible range: 9–45), because quiet quitting was analyzed as a single overall outcome in the regression and mediation models; subscale scores were not analyzed. In the Turkish adaptation study, the Cronbach’s alpha internal consistency coefficient of the scale was reported as 0.68. Based on the data from this study, the Cronbach’s alpha value for this scale was determined to be 0.71.

#### Career decision regret scale

The Career Decision Regret Scale was developed by Brehaut et al. (2003) and adapted into Turkish by Erdurcan and Kırdök (2017). The items refer to regret about the individual’s career (occupational) decision. The scale has a unidimensional structure and consists of five items. It is rated on a 5-point Likert-type scale ranging from “0 = Strongly disagree” to “4 = Strongly agree.” Items 1, 3, and 5 in the Turkish version of the scale are reverse-coded. The scores obtained from the scale are calculated based on the total score (possible range: 0–20), with higher scores indicating higher levels of career decision regret. In the adaptation study conducted by Erdurcan and Kırdök (2017), the Cronbach’s alpha internal consistency coefficient of the scale was reported as 0.91, whereas the Cronbach’s alpha coefficient was found to be 0.88 in the present study.

### Data analysis

The data were analyzed using SPSS version 25.0 and the PROCESS Macro (v4.2) developed by Hayes (2022). First, frequency analyses and descriptive statistics were conducted. The normality of the data distribution was examined using the Kolmogorov–Smirnov test, which indicated that the data were not normally distributed (*p* < 0.05). With a sample of 646, this test is sensitive to small departures from normality, and its result alone does not show that the deviations were substantial. Non-parametric tests (the Mann–Whitney U test, the Kruskal–Wallis test, and Spearman’s correlation) were used for the group comparisons and bivariate associations. The hierarchical regression and the PROCESS model are based on ordinary least squares, and the indirect effect was evaluated with bootstrap confidence intervals, which do not assume a normal sampling distribution. Following significant Kruskal–Wallis tests, pairwise comparisons were examined to determine which groups accounted for the significant differences.

Effect sizes were calculated from the reported test statistics. For the Mann–Whitney U tests, the rank-biserial correlation was computed as r_rb_ = 1 − 2 U/(n_1_ × n_2_), where U is the smaller of the two U values. For the Kruskal–Wallis tests, epsilon-squared was computed as ε^2^ = H/(N − 1) (Tomczak and Tomczak, 2014). As descriptive benchmarks, values of about 0.10, 0.30, and 0.50 for r_rb_ and about 0.01, 0.06, and 0.14 for ε^2^ were taken to indicate small, medium, and large effects, respectively.

According to Büyüköztürk (2018), correlation coefficients with absolute values ranging from 0.70 to 1.00 indicate a high level of association, values ranging from 0.30 to 0.70 indicate a moderate level of association, and values ranging from 0.00 to 0.30 indicate a low level of association. These criteria were used to interpret the results of the Spearman correlation analysis.

The internal consistency of the measurement instruments used in the study was assessed using Cronbach’s alpha reliability coefficient. After the demographic variables were entered as covariates, the associations of job-related emotional exhaustion and career decision regret with quiet quitting were examined using hierarchical regression analysis. The statistical mediating role of career decision regret in the association between job-related emotional exhaustion and quiet quitting was tested using PROCESS Macro Model 4 with 5,000 bootstrap samples and a 95% confidence interval, with age entered as a covariate. In line with the cross-sectional design, the terms total, direct, and indirect effect are used below only in their statistical sense and do not imply causal effects. The potential multicollinearity among the variables was assessed by examining variance inflation factor (VIF) values.

## Results

As shown in Table 2, the participants’ mean score on the Job-Related Emotional Exhaustion Scale was 13.96 ± 4.65, the mean score on the Quiet Quitting Scale was 26.58 ± 4.92, and the mean score on the Career Decision Regret Scale was 11.38 ± 3.11.

**Table 2:** Descriptive statistics for the scales.

Scale	N	Min	Max	Median	Mean	Std. deviation
Job-related emotional exhaustion scale	646	3	24	14.00	13.96	4.652
Quiet quitting scale	646	17	41	26.00	26.58	4.922
Career decision regret scale	646	4	19	12.00	11.38	3.113

As shown in Table 3, no statistically significant differences were found in job-related emotional exhaustion (U = 47538.0; *p* = 0.074) or quiet quitting scores (U = 51682.5; *p* = 0.979) according to gender. However, career decision regret scores were significantly higher among male participants than among female participants (U = 42210.0; *p* < 0.001; r_rb_ = 0.18). Regarding marital status, no statistically significant differences were found in job-related emotional exhaustion (U = 35051.5; *p* = 0.321) or career decision regret scores (U = 36617.0; *p* = 0.836). In contrast, quiet quitting scores were significantly higher among married participants than among single participants (U = 30049.5; *p* < 0.001; r_rb_ = 0.19). Regarding employment type, job-related emotional exhaustion scores were significantly higher among part-time employees than among full-time employees (U = 45412.0; *p* = 0.013; r_rb_ = 0.11), whereas career decision regret scores were significantly higher among full-time employees than among part-time employees (U = 46532.5; *p* = 0.045; r_rb_ = 0.09). No statistically significant difference was found in quiet quitting scores according to employment type (U = 49442.0; *p* = 0.454). The effect sizes of the significant contrasts were small (r_rb_ = 0.09–0.19).

**Table 3:** Analyses of gender, marital status, and employment type variables.

Scale	Gender	n	Mean rank	Sum of ranks	U	*p*
Job-related emotional exhaustion scale	Female	352	335.45	118078.00	47538.000	0.074
	Male	294	309.19	90903.00		
Quiet quitting scale	Female	352	323.67	113933.50	51682.500	0.979
	Male	294	323.29	95047.50		
Career decision regret scale	Female	352	296.41	104338.00	42210.000	**< 0.001**
	Male	294	355.93	104643.00		
	Marital status					
Job-related emotional exhaustion scale	Married	149	336.76	50176.50	35051.500	0.321
	Single	497	319.53	158804.50		
Quiet quitting scale	Married	149	370.33	55178.50	30049.500	**< 0.001**
	Single	497	309.46	153802.50		
Career decision regret scale	Married	149	320.75	47792.00	36617.000	0.836
	Single	497	324.32	161189.00		
	Employment type in the sports sector					
Job-related emotional exhaustion scale	Part-time	279	344.23	96041.00	45412.000	0.**013**
	Full-time	367	307.74	112940.00		
Quiet quitting scale	Part-time	279	317.21	88502.00	49442.000	0.454
	Full-time	367	328.28	120479.00		
Career decision regret scale	Part-time	279	306.78	85592.50	46532.500	0.**045**
	Full-time	367	336.21	123388.50		

Rank-biserial correlations (r_rb_ = 1 − 2 U/(n_1_ × n_2_)) for the significant contrasts: career decision regret by gender = 0.18; quiet quitting by marital status = 0.19; job-related emotional exhaustion by employment type = 0.11; career decision regret by employment type = 0.09. Bold values indicate statistically significant results (*p* < 0.05).

As shown in Table 4, a statistically significant difference was found among the age groups in terms of job-related emotional exhaustion scores (H = 39.550; *p* < 0.001; ε^2^ = 0.061). The pattern was non-linear: participants aged 28–37 had the lowest mean rank (247.17), below that of participants aged 19–27 (324.99), whereas participants aged 38 years and older had the highest mean rank (414.11); all three pairwise differences were significant. A statistically significant difference was also found among the age groups in terms of quiet quitting scores (H = 36.003; *p* < 0.001; ε^2^ = 0.056), with participants aged 38 years and older having higher scores than those in the other age groups; the 28–37 group again had the lowest mean rank (283.82), although its difference from the 19–27 group (312.41) was not significant. A significant difference was likewise observed in career decision regret scores across age groups (H = 7.540; *p* = 0.023; ε^2^ = 0.012), with participants aged 28–37 having higher scores than those aged 19–27. Regarding duration of employment in the sports sector, a statistically significant difference was found in job-related emotional exhaustion scores (H = 37.746; *p* < 0.001; ε^2^ = 0.059). The difference was attributable to participants with 11–15 years and 16 years or more of employment having higher scores than those with 1–5 and 6–10 years of employment; the mean rank was highest in the 11–15 years group (430.02) and lowest in the 6–10 years group (255.87). A significant difference was also found among the groups in terms of quiet quitting scores (H = 14.169; *p* = 0.003; ε^2^ = 0.022), with participants who had 16 years or more of employment having higher scores than those with 1–5 years of employment. A statistically significant difference was also found in career decision regret scores according to duration of employment (H = 44.229; *p* < 0.001; ε^2^ = 0.069). Participants with 11–15 years of employment (mean rank = 457.65) had higher scores than each of the other groups, and participants with 1–5 years of employment (317.83) had higher scores than those with 6–10 years (253.30). By the benchmarks given above, the effect sizes were small for career decision regret by age and for quiet quitting by duration of employment (ε^2^ = 0.012 and 0.022) and close to the medium benchmark for the remaining comparisons (ε^2^ = 0.056–0.069).

**Table 4:** Analyses of age and duration of employment in the sports sector variables.

Scale	Age	n	Mean rank	H	*p*	The source of the difference
Job-related emotional exhaustion scale	19–27 years^a^	447	324.99	39.550	**< 0.001**	a-b a-c b-c
	28–37 years^b^	112	247.17			
	38 years and older^c^	87	414.11			
Quiet quitting scale	19–27 years^a^	447	312.41	36.003	**< 0.001**	a-c b-c
	28–37 years^b^	112	283.82			
	38 years and older^c^	87	431.56			
Career decision regret scale	19–27 years^a^	447	310.16	7.540	0.**023**	a-b
	28–37 years^b^	112	354.73			
	38 years and older^c^	87	351.84			
	Duration of employment in the sports sector					
Job-related emotional exhaustion scale	1–5 years^a^	474	313.66	37.746	**< 0.001**	a-c a-d b-c b-d
	6–10 years^b^	72	255.87			
	11–15 years^c^	63	430.02			
	16 years or more^d^	37	399.78			
Quiet quitting scale	1–5 years^a^	474	310.60	14.169	0.**003**	a-d
	6–10 years^b^	72	322.65			
	11–15 years^c^	63	371.90			
	16 years or more^d^	37	408.03			
Career decision regret scale	1–5 years^a^	474	317.83	44.229	**< 0.001**	a-b a-c b-c c-d
	6–10 years^b^	72	253.30			
	11–15 years^c^	63	457.65			
	16 years or more^d^	37	304.35			

Superscript letters identify the groups compared in the last column. ε^2^ = H/(N − 1). Age: job-related emotional exhaustion = 0.061, quiet quitting = 0.056, career decision regret = 0.012. Duration of employment: job-related emotional exhaustion = 0.059, quiet quitting = 0.022, career decision regret = 0.069. Bold values indicate statistically significant results (*p* < 0.05).

Table 5 presents the results of the correlation analysis examining the relationships among job-related emotional exhaustion, quiet quitting, and career decision regret. A positive and statistically significant relationship was found between job-related emotional exhaustion and quiet quitting (*r* = 0.474; *p* < 0.001). A positive and statistically significant relationship was also found between job-related emotional exhaustion and career decision regret (*r* = 0.253; *p* < 0.001). Furthermore, a positive and statistically significant relationship was found between quiet quitting and career decision regret (*r* = 0.344; *p* < 0.001).

**Table 5:** Correlation analysis between scales.

Scale	Statistic	Job-related emotional exhaustion scale	Quiet quitting scale	Career decision regret scale
Job-related emotional exhaustion scale	*r*	1		
	*p*			
Quiet quitting scale	*r*	0.474^**^	1	
	*p*	< 0.001		
Career decision regretscale	*r*	0.253^**^	0.344^**^	1
	*p*	< 0.001	< 0.001	

**p < 0.01.

Table 6 presents the results of the hierarchical regression analysis examining the variables predicting participants’ levels of quiet quitting. In Model 1, gender (*β* = −0.004; *p* = 0.914), marital status (*β* = 0.002; *p* = 0.969), employment type (*β* = −0.084; *p* = 0.059), and duration of employment in the sports sector (*β* = −0.015; *p* = 0.831) did not significantly predict quiet quitting, whereas age was found to be a positive and significant predictor (*β* = 0.176; *p* = 0.010). Because age and duration of employment were each entered as a single coded variable, which assumes a linear trend, and Table 4 shows non-linear patterns, these coefficients should be interpreted with caution. When job-related emotional exhaustion was entered into the model in Model 2, it was found to positively and significantly predict quiet quitting (*β* = 0.492; *p* < 0.001). The proportion of explained variance increased in Model 2, reaching R^2^ = 0.266. In Model 3, following the addition of career decision regret, this variable was also found to positively and significantly predict quiet quitting (*β* = 0.297; *p* < 0.001). In the final model, the proportion of explained variance increased to R^2^ = 0.342 (Table 7).

**Table 6:** Hierarchical regression analysis for predicting quiet quitting.

Model	Predictor	B	Std. error	β	t	*p*	VIF
1	(Constant)	25.403	1.691		15.025	< 0.001	
	Gender	−0.042	0.388	−0.004	−0.108	0.914	1.026
	Marital status	0.023	0.593	0.002	0.039	0.969	1.717
	Employment type in the sports sector	−0.835	0.441	−0.084	−1.893	0.059	1.316
	Age	1.204	0.465	0.176	2.591	0.010	3.071
	Duration of employment in the sports sector	−0.083	0.390	−0.015	−0.213	0.831	3.305
2	Job-related emotional exhaustion scale	0.520	0.037	0.492	13.964	< 0.001	1.077
3	Career decision regret scale	0.470	0.055	0.297	8.589	< 0.001	1.159

Model summary	R^2^	Adjusted R^2^	ΔR^2^	F Change	*p*
Model 1	0.041	0.032	0.041	4.559	< 0.001
Model 2	0.266	0.257	0.224	194.990	< 0.001
Model 3	0.342	0.333	0.076	73.766	< 0.001

**Table 7:** Statistical mediation by career decision regret of the association between job-related emotional exhaustion and quiet quitting.

Pathways of effect	*B (Coefficient)*	Standard error	t	*p*	95% CI (LLCI)	95% CI (ULCI)
Total effect (c Path)						
Emotional Exhaustion → Quiet Quitting	0.5096	0.0359	14.1843	< 0.001	0.4391	0.5802
Direct effect (c′ Path)						
Emotional Exhaustion → Quiet Quitting	0.4275	0.0355	12.0425	< 0.001	0.3578	0.4972
Indirect Effect (a × b Path)	B (Coefficient)	BootSE			BootLLCI	BootULCI
Emotional Exhaustion → Career Decision Regret → Quiet Quitting	0.0821	0.0187			0.0481	0.1218

CI, confidence interval; LLCI and ULCI, lower and upper limits; bootstrap estimates are based on 5,000 samples. Age was included as a covariate. “Effect” denotes a statistical model term and does not imply causation.

To test whether career decision regret statistically mediates the association between job-related emotional exhaustion and quiet quitting, a Model 4 mediation analysis was conducted, with participants’ age included in the model as a covariate. The findings indicated that, before the mediator (career decision regret) was entered into the model, the total effect of job-related emotional exhaustion on quiet quitting (c path) was positive and statistically significant (B = 0.5096; t = 14.184; *p* < 0.001). After the mediator was included in the model, the direct effect of job-related emotional exhaustion on quiet quitting (c′ path) remained statistically significant, although its magnitude decreased (B = 0.4275; t = 12.042; *p* < 0.001). The indirect effect through career decision regret (a × b path; H4) was statistically significant (B = 0.0821; BootSE = 0.0187); the 95% confidence interval based on 5,000 bootstrap samples did not include zero (95% CI [0.0481, 0.1218]). Since the direct effect remained significant after the mediator was included in the model, the result corresponds to partial statistical mediation. The indirect effect was modest: as a descriptive ratio, it amounted to about 16.1% of the total effect (0.0821/0.5096), and this ratio is not interpreted as a causal proportion mediated. The finding indicates that higher job-related emotional exhaustion was associated with higher career decision regret, which in turn was associated with higher quiet quitting. This pattern is consistent with the proposed process but, given the cross-sectional design, cannot establish temporal or causal ordering.

## Discussion

This study examined whether job-related emotional exhaustion and career decision regret are associated with quiet quitting among private sports facility employees and whether career decision regret statistically mediates the association between job-related emotional exhaustion and quiet quitting. The four hypotheses were supported in the statistical sense: job-related emotional exhaustion was positively associated with quiet quitting (H1) and with career decision regret (H2), career decision regret was positively associated with quiet quitting (H3), and the indirect association through career decision regret was significant (H4). The mediation was partial and modest. The direct association remained strong (B = 0.4275), and the indirect association (B = 0.0821) corresponded to about 16.1% of the total as a descriptive ratio.

The positive association between job-related emotional exhaustion and quiet quitting is in line with COR reasoning, in which employees whose resources are depleted limit further investment in order to protect their remaining resources (Hobfoll et al., 2018). It is also consistent with studies reporting that emotional exhaustion is related to lower commitment, turnover, and psychological withdrawal from work (Lapointe et al., 2011; Fernet et al., 2012; Du Plooy and Roodt, 2010; Demerouti et al., 2010; Bolton et al., 2012) and with the association between emotional exhaustion and quiet quitting reported among healthcare workers (Gökkaya, 2024). In private sports facilities, where instructors sustain emotional labour in continuous contact with members (Maguire, 2001; Lee, 2023; Roderick et al., 2017), restricting effort to the formal requirements of the role may be one way in which exhausted employees conserve resources. The consequences of exhaustion have been particularly emphasized in work environments characterized by high stress, heavy workloads, and continuous performance pressure (Maslach and Leiter, 2022; Rollins et al., 2021; Maslach and Goldberg, 1998; West et al., 2018; Hurria, 2023).

Career decision regret statistically accounted for a small part of this association. The literature also indicates that regret regarding career decisions is related to lower job satisfaction, organizational commitment, and occupational satisfaction and to turnover-related cognitions (Young et al., 2023; Cheval et al., 2019; Ergün, 2022; Verbruggen and Van Emmerik, 2020; Polat and Yeşil, 2025). The present finding suggests that career decision regret may represent one cognitive-evaluative pathway linking depleted resources to minimum-role withdrawal. However, most of the association between job-related emotional exhaustion and quiet quitting was not statistically accounted for by regret, so other processes that were not measured here are likely to be involved. Evidence from sport-science students indicates that self-efficacy is related to coping-with-stress behaviors, supporting the inclusion of personal psychological resources in future models of work-related withdrawal in sport settings (Toktaş et al., 2022). Related evidence from physically active adults has shown that perceived stress is negatively associated with psychological resilience and quality of life, further highlighting the relevance of psychological resources to well-being in sport and exercise contexts (Ulukan et al., 2026).

These results are consistent with, but do not prove, the proposed process. Because all variables were measured at one time point, alternative orderings cannot be excluded. Employees who regret their career choice may experience their work as more exhausting, quiet quitting may itself be related to later exhaustion or regret, and the three variables may share unmeasured common causes such as working conditions or organizational support. Statistical mediation in cross-sectional data cannot establish temporal or causal ordering, and longitudinal designs are needed to test the direction of these associations.

In the hierarchical regression, job-related emotional exhaustion and career decision regret each statistically predicted quiet quitting after the demographic variables had been entered, and the explained variance rose from 4.1 to 34.2%. Among the demographic variables, only age was a significant predictor. Because age and duration of employment were entered as single coded variables although their bivariate patterns were non-linear, the covariate coefficients should be interpreted with caution.

The demographic comparisons were secondary and exploratory, and their effect sizes were small or close to the medium benchmark. Men reported higher career decision regret than women, whereas job-related emotional exhaustion and quiet quitting did not differ by gender. The latter result is in line with reports that exhaustion and quiet quitting are related more closely to working conditions than to gender (Almarzoqee et al., 2026; Park et al., 2026; Seidler et al., 2014; Chiara et al., 2019; Gellock and Dwyer, 2023; Snarr and Beasley, 2022). Career-advancement expectations and financial responsibilities have been described among male nurses (Wu et al., 2025), and career preparation and employment opportunities have been discussed for careers in sport (Martin et al., 2026; Hutchinson, 2009). However, these factors were not measured in the present study, and part of this evidence comes from a different occupational group; they are therefore offered only as possible explanations requiring future testing. Married employees reported higher quiet quitting than single employees. Work–family conflict has been related to work withdrawal (Choi, 2024) and has been discussed in relation to work–life balance and sport settings (Sirgy and Lee, 2018; Brough et al., 2022; Ni et al., 2022; Dixon and Bruening, 2005), but it was not measured here and remains a possible explanation requiring future testing. Part-time employees reported higher job-related emotional exhaustion, and full-time employees reported higher career decision regret. Variable working hours, job insecurity, and differing responsibilities are possible explanations (Seibt and Kreuzfeld, 2021; Conway and Briner, 2002; Bentzen et al., 2020), but income, job security, and role ambiguity were not assessed in this study.

The age and duration-of-employment patterns were not linear. For both job-related emotional exhaustion and quiet quitting, the 28–37 age group had the lowest mean ranks, below those of the youngest group, and the oldest group had the highest; career decision regret was higher in the 28–37 group than in the 19–27 group. This pattern does not support a simple increase with age. It agrees with studies reporting that the relationship between age and burnout is not linear and depends on working conditions and experience (Gómez-Urquiza et al., 2017; García-Arroyo and Osca, 2017; Brewer and Shapard, 2004; Ramos et al., 2016). For duration of employment, job-related emotional exhaustion was higher in the 11–15 years and 16 years or more groups than in the two shorter-tenure groups, quiet quitting was highest in the longest-tenure group, and career decision regret peaked in the 11–15 years group, whereas the 6–10 years group had the lowest mean ranks for exhaustion and regret. Earlier studies of coaches and physical education teachers have likewise reported that experience is related to burnout, although not consistently across samples (Dükel and Pehlevan, 2021; Öztürk, 2018; Gencay and Gencay, 2011; Brouwers et al., 2011; Gençay, 2007; Smith and Leng, 2003; Aronsson et al., 2017). Career stage may contribute to these differences (Van der Horst et al., 2017; Ng and Feldman, 2009), but this was not tested. The older and longer-tenure groups were small (n = 37–87), so these comparisons should be interpreted with caution.

### Practical implications

The findings point to several areas that managers of private sports facilities may consider. Because the design is cross-sectional, these are suggestions derived from observed associations and not evidence that such measures are effective.

- Part-time employees reported higher job-related emotional exhaustion. Predictable scheduling, clear contract terms, and manageable session loads and client-contact time may be particularly relevant for this group.
- Full-time employees reported higher career decision regret. Visible career pathways and professional development opportunities may be relevant for full-time staff.
- Job-related emotional exhaustion was higher among employees with longer tenure, particularly those with 11–15 years of employment, who also reported the highest career decision regret, and quiet quitting ranks were highest among those with 16 years or more. Workload review, supervisor support, and renewed development opportunities may be considered for mid- and late-career employees.
- Because job-related emotional exhaustion showed the strongest association with quiet quitting, periodic assessment of employee well-being may help facilities to notice exhaustion early.

### Strengths and limitations

A strength of this study is its relatively large sample of employees from several occupational roles in private sports facilities, a group that has seldom been studied in relation to quiet quitting. Several limitations should be considered. First, the design was cross-sectional, so no temporal or causal inference can be drawn, and the mediation result is statistical only. Second, participants were recruited by convenience sampling from private sports facilities in four districts of two provinces (Sapanca and Serdivan in Sakarya; Başiskele and İzmit in Kocaeli), which limits the generalizability of the findings to other regions, to public facilities, and to other parts of the sports sector such as clubs and federations. Third, all variables were measured by self-report in a single session, so common-method bias may have inflated the observed associations, and no statistical check for common-method variance is reported. Fourth, the sample was skewed toward younger and short-tenure employees (69.2% aged 19–27 years; 73.4% with 1–5 years of employment), and some subgroups were small (e.g., *n* = 37 with 16 years or more of employment), which reduces the precision of the subgroup comparisons. Fifth, the distribution of participants across occupational roles is not reported, and emotional demands may differ between administrative and instructional roles. Sixth, age and duration of employment were entered in the regression as single coded variables despite non-linear bivariate patterns, and the mediation model included only age as a covariate; the results may therefore be sensitive to model specification. Seventh, current-sample reliability is reported only for the Career Decision Regret Scale, the Quiet Quitting Scale was analyzed as a total score although the original scale has three factors, and its reliability in the adaptation study was modest (0.68). Finally, regression diagnostics and a formal sample-size justification are not reported.

## Conclusion

Among employees of private sports facilities in four districts of Sakarya and Kocaeli, job-related emotional exhaustion was positively associated with quiet quitting, and career decision regret showed partial and modest statistical mediation of this association. In the secondary analyses, career decision regret was higher among men and full-time employees, quiet quitting was higher among married employees, job-related emotional exhaustion was higher among part-time employees, and all three variables differed across age and duration-of-employment groups in non-linear patterns. The findings are consistent with a resource-loss account of quiet quitting but, being cross-sectional, do not establish temporal or causal ordering and apply only to the sampled private sports facilities.

The following recommendations for future research are proposed based on the findings of the study:

- Similar studies can be conducted in other regions, in public facilities, and in other parts of the sports sector.
- Future studies can examine the relationships among variables by incorporating additional variables such as organizational commitment, job satisfaction, psychological resilience, perceived organizational support, and leadership into the research model. Psychological resilience has also been examined as a relevant resource under demanding sport conditions, supporting its inclusion in future models of employee well-being in the sports sector (Çağlar et al., 2025).
- Longitudinal and experimental research designs can be employed to test the temporal ordering of job-related emotional exhaustion, career decision regret, and quiet quitting.

## Data Availability

The original contributions presented in the study are included in the article/supplementary material, further inquiries can be directed to the corresponding author.
